# Editorial: Multimodal communication and multimodal computing

**DOI:** 10.3389/frai.2023.1234920

**Published:** 2023-06-27

**Authors:** Alexander Mehler, Andy Lücking, Tiansi Dong

**Affiliations:** ^1^Text Technology Lab, Goethe-University Frankfurt, Frankfurt, Germany; ^2^Laboratoire de Linguistique Formelle (LLF), Université Paris Cité, Paris, France; ^3^Neurosymbolic Representation Learning Group, Fraunhofer IAIS, Sankt Augustin, Germany

**Keywords:** human-object interactions (HOIs), multimodal learning and analytics, visual-linguistic interaction, text-image analysis, unified methodology

After a successful and text-centered period, AI, computational linguistics, and natural language engineering need to face the “ecological niche” (Holler and Levinson, 2019) of natural language use: *face-to-face interaction*. A particular challenge of human processing in face-to-face interaction is that it is fed by information from the various sense modalities: it is *multimodal*. When talking to each other, we constantly observe and produce information on several channels, such as speech, facial expressions, hand-and-arm gestures, and head movements. To learn drive, we first learn theories about traffic rules in driving schools. After passing the examinations, we practice on the streets, accompanied by an expert sitting aside. We ask questions and follow instant instructions from this expert. These symbolic traffic rules and instant instructions shall be quickly and precisely grounded to the perceived scenes, with which the learner shall update and predict other cars behaviors quickly, then determine her/his own driving action to avoid potential dangers. As a consequence, multimodal communication needs to be *integrated* (in perception) or *distributed* (in production). This, however, characterizes multimodal computing in general (but see also Parcalabescu et al., 2021). Hence, AI, computational linguistics and natural language engineering that address multimodal communication in face-to-face interaction have to involve multimodal computing–giving rise to the next grand research challenge of those and related fields. This challenge applies to all computational areas which look beyond sentences and texts, ranging from interacting with virtual agents to the creation and exploitation of multimodal datasets for machine learning, as exemplified by the contributions in this Research Topic.

From this perspective, we face several interwoven challenges: On the one hand, AI approaches need to be informed about the principles of multimodal computing to avoid simply transferring the principles of Large Language Models to multimodal computing. On the other hand, it is important that more linguistically motivated approaches do not underestimate the computational reconstructability of multimodal representations. They might otherwise have to share experiences with parts of computational linguistics, given the success of models such as OpenAI's ChatGPT (cf.Wolfram, 2023), which confronted them with the realization that even higher-order linguistic annotations could be taken over by digital assistants and consequently render the corresponding linguistic modeling work obsolete. Again, the scientific focus on face-to-face communication seems to point to a middle ground. This is because we are dealing with the processing of highly contextualized data whose semantics require recourse to semantic or psycholinguistic concepts such as utterance situation Schüz et al., situation models or mental models (Johnson-Laird, 2010; Ragni and Knauff, 2013; Alfred et al., 2020) or reference to concepts such as grounding (Harnad, 1990), for the automatic reconstruction of which there are not yet adequate computer-based approaches, certainly not on the basis of scenarios such as one-shot or few-shot learning, since the corresponding experiential content is not available as (annotated) mass data. The particular moment in which one finds oneself information-theoretically at this point can be formulated as follows: large domains of linguistic and multimodal interactions, if they provide a sufficient number of patterns for association learning, are well manageable with methods based on current neural networks. However, as soon as we go beyond such associative regularities and arrive at a kind of meaning constitution that includes the *about* of communicative interaction—when we are dealing, so to speak, with the alignment of immediate objects and interpretants in the sense of Peirce (1934) (cf.Gomes et al., 2007 for a reference to Peirce in AI)—we reach the limits of such models, which have by no means already been explored and which we believe we can identify once again in the area of face-to-face communication. It is obvious that AI models need to complement bottom-up approaches with top-down approaches that start from multimodal situation models grounded in face-to-face communication, or at least from the notion of discourse as put forward by Alikhani et al., an approach that finds its obvious extension in an approach more oriented to terms of social science (see, for example, Cheema et al.).

From another angle, AI applications are increasingly appearing in complex communication situations or action contexts as quasi-agentive fourth-generation interfaces (Floridi, 2014), which raises the question of their status with respect to the distinction between simulation, emulation, and realization (Pattee, 1989). Looking again at the driving example, the issue here is that AI applications are increasingly applied in real-world contexts, where their use is contextualized each time by corresponding multimodal real-world data, representing a potential grounding-relevant resource that can be re-used for fine-tuning such models or even grounding them. One could object that such an AI agent is nothing more than a simulation, which in principle cannot know anything about this its status. However, such simulations perform under real conditions in interaction with more and more humans in no longer simulatively closed systems [of agent(s) and environment(s)], and this can drive a technological development of these systems in terms of life-long learning, which can ultimately make them appear as *realizations* of *interaction partners*. But here, too, one can ask what the limits of this interaction are, even if it is multimodal. For it is something fundamentally different to process multimodally generated data than to experience it through independent production, of which the notion of telic affordance provides a vivid example, since it is based on people's habits of use, a kind of use that AI systems are mostly incapable of at present. Is it this kind of difference, such as being able to identify a telic affordance either through one's own use or merely by observing data left by uses of human agents, that constitutes one of the limitations implied above? Be that as it may, in their paper Henlein et al. explore the question of the learnability of affordances using vision-based AI models, an approach that we argue could also be interpreted as an example of measuring the implied limit(s).

The counter-scenario to agents interacting with us as artificial interactors in real-world environments is a completely virtualized scenario in which both human and artificial agents interact as avatars (see Chalmers, 2022). Here, conversely, it is the human who enters the sphere of simulation, so to speak, rather than the simulation that we encounter as a putative realization. The key research advantage of such settings is that the resulting multimodal data becomes largely amenable to direct digitization and thus automatic analysis. This concerns areas as diverse as speech data, data regarding interaction with objects, lip movement data, facial expression data, eye movement data, head movement data, manual gesture data, body movement data, and (social) space-related behavioral data, as well as (social) distance behavioral data (see Mehler et al., 2023 for a corresponding formal data model in the context of VR). Evidently, virtual worlds provide an excellent experimental environment for the study of artificial interaction. This is addressed in the work of Nunnemann et al.. It can be seen as an example of the study of grounding issues that directly affect the actors involved and thus relate to the issue of grounding interactions. This raises the broader question of how to advance semantic theories that can be experimentally falsified, as VR systems seem to fit into the paradigm of an Experimental Semiotics (Galantucci and Garrod, 2011) in exemplary way, a fit that could not have been foreseen even just a few years earlier. In other words: in VR, the research strands of face-to-face communication, dialogic communication (Galland et al.), multimodal information processing, grounding in interaction environments that may be equipped with artifacts of a wide variety of affordances, and 4th-order artificial interaction (Floridi, 2014) seem to come together in exemplary fashion, suggesting much further research in this direction in the future. The time is ripe for a fundamental expansion of the empirical base of linguistics and communication studies research that knows how to utilize the possibilities of AI-based systems experimentally for its research purposes, and conversely, for the acquisition of ever more extensive multimodal data for the situation-specific grounding of AI systems, which will ideally no longer rely solely on text windows and wordpiece or subword analogies (Song et al., 2021) (cf. the *Bag-of-Visual Words* approach of Bruni et al., 2014) to infer the putative underlying semantics from the associations shadowed in the character strings observable by means of these windows. At present, it is unclear how far this line of research is developed or to what extent other than the current greedy segmentation models or tokenizers are already emerging that can also identify multimodal ensembles as recurrent data units. Nevertheless, as in the case of transformers (Devlin et al., 2019), this line of research can point to a worthwhile direction for development.

A crucial part of the multimodal challenge is to address the question of how to assemble, let alone parse, multimodal representations. A successful multimodal system shall unify representations from different channels. The fundamental challenge is to merge the two complementary modals, namely, the neural modal and the symbolic modal, and be capable of solving problems from both perspectives (Dinsmore, 1992). Geometrical structure is advocated as a potential cognitive representation apart from symbols or neural-networks (Gärdenfors, 2000). A recent geometric approach successfully unified large symbolic tree structures with pre-trained vector embedding precisely (Dong, 2021), and opens a new door to allow symbolic structures to have precise neural representation, and potentially remove the gap between neural modal and symbolic modal (Bechtel and Abrahamsen, 2002; Dong et al., 2022; Sun, 2023).

Multimodal representations can be compared to musical scores where the different “voices” co-occur and may (or not) be tied together by relevance (Lücking and Ginzburg, 2023) (see Mehler and Lücking, 2009 for an example and a formalization of such kinds of representations). In this respect, McNeill (1992) and Kendon (2004) have shown in seminal works that manual gesture and speech form unified messages, but without specifying systematic, computational means for analyzing multimodal utterances. Alikhani et al. argue in their contribution “*Image–text coherence and its implications for multimodal AI*” that the appropriate level for processing multimodal representations in AI is the level of *discourse*. By example of image–text pairs, they apply *coherence theory* to capture the structural, logical and purposeful relationships between images and their captions. Using a dataset of image–text coherence relations, the authors question whether simple coherence markers are accounted for in two pre-trained multimodal language models, CLIP (Radford et al., 2021) and ViLBERT (Lu et al., 2019). Alikhani et al. move on to use these results to critique and improve the architecture of machine learning models, and to develop coherence-based evaluations of multimodal AI systems.

Image–text relations are also investigated by Cheema et al.. The authors focus on the relation between images and texts in the setting of news. They propose directions for multimodal learning and analytics in social sciences. Taking a largely semiotic perspective, the authors bring together news value analysis of news media from both a production and reception perspective, and the multimodality of news articles in terms of image–text relations which go beyond (related to the coherence-driven approach by Alikhani et al.) mere captions. The framework is applied to a couple of examples and is intended to shape larger-scale machine learning applications in the context of multimodal media analysis, as exemplified by means of a number of potential uses cases.

Turning from two-dimensional pictures to objects within virtual reality, Henlein et al. present their research on Human-Object Interaction (HOI) and augment the HICO-DET dataset (Chao et al., 2018) to distinguish Gibsonian (Gibson, 1979, Chap. 8) affordances (actions to which objects “invite”) and telic affordances (objects' conventionalized purposes) (Pustejovsky, 2013). They successfully train the computational model AffordanceUPT on their extended resource and show that is is able to distinguish intentional use from Gibsonian exploitation, even for new objects. Hence, Henlein et al. contribute to a better understanding of clustering of objects according to their action potentials, in particular a clustering between perceptual features and intention recognition.

(Virtual) Objects and characters are potential referents in human–human and human–computer interaction. Nunnemann et al. investigate “*The effects of referential gaze in spoken language comprehension: human speaker vs. virtual agent listener gaze*”. Hence, they address multimodal computing at the interface of human and artificial communication: On the one hand, people are known to respond to virtual agent gaze (Ruhland et al., 2015). On the other hand, during referential processing eye movements to objects in joint visual scenes are closely time locked to referring words used to describes those scenes (Eberhard et al., 1995). Using eye-tracking methods Nunnemann et al. compared the influence of human speaker gaze to that of virtual agent listener gaze in sentence verification tasks. While they could replicate findings that participants draw on human speaker gaze, they do not rely on the gaze of the virtual agent. Thus, the study hints at important directions in the creation of and interaction with virtual agents, pointing out the influence of the communicative role of virtual agents (i.e., speaker vs. hearer) and potentially the need of a Theory of Mind (Krämer, 2005).

While gaze can be used for establishing reference (in particular in dangerous situations, see Hadjikhani et al., 2008), the most important linguistic devices for referring are verbal referring expressions. The form of these referring expressions is adapted to the utterance situation: Schüz et al. discuss the representation problem in the sub-field of Referring Expression Generation (REG), where expressions are depended on contexts. They provide a systematic review of a variety of visual contexts and approaches to REGs, and strongly argue for an integrated or unified perspective or methodology. The focus is on different input modalities and how they shape the information that is needed for successful reference (i.e., enable the addressee to single out the intended object), thereby complementing and going beyond established research on multimodal deictic output (e.g., Kranstedt et al., 2006; van der Sluis and Krahmer, 2007).

In conversation, interlocutors exhibit conversational strategies or styles (Tannen, 1981). Galland et al. explore communicative preferences in the context of human-computer interaction in terms of task-oriented and socially-oriented dialogue acts. By utilizing reinforcement learning, they train an artificial agent to adapt its strategy to meet the preference of a human user by combining task-oriented and socially-oriented dialog act. This is achieved by combining four components: an engagement estimator (mainly based on the user's non-verbal behavior), a topic manager (keeping track of the user's favorite topics), a conversational preferences estimator (estimates the user's task/social preference a each turn), and a dialog manager (selects the most appropriate turn according to the artificial agent's user model). Subjective experiments involving over 100 participants show a cross-modal influence: adapting to a user's preferred conversational strategy or style affects the human's perception and increases user engagement.

The Research Topic *Multimodal communication and multimodal computing* comprises six different contributions that highlight different areas and challenges of the interplay between communication and computing, as they have emerged not only due to the recent rapid development of AI methods. What unites these contributions is their common focus on multimodality, which, however, they treat from very different perspectives: be it in terms of text-image relations, the affordances detectable through images, the interaction between humans and artificial agents, or the specific status of referring expressions in spoken language comprehension. From a methodological perspective, these approaches are interesting because they redirect the AI focus from Big Data to Small or even Tiny Data, massively emphasizing the situatedness of communication in its multiple multimodal manifestations. What we ultimately lack, however, is an approach that integrates these heterogeneous research directions and their underlying distributed data resources to ground a more comprehensive multimodal semantics in a final joint research effort by linguistics, computational linguistics, and computer science—before this will all be taken over by AI agents.

## Author contributions

This Research Topic on *Multimodal communication and multimodal computing* was proposed by AM, AL, and TD. The editors worked collaboratively to decide which potential authors to invite and which papers were accepted or rejected. The single manuscripts were subject to review by the corresponding handling editor as well as peer reviewers. This editorial was drafted by AM, AL, and TD. All authors contributed to the article and approved the submitted version.
